# Clinical outcomes of immune checkpoint inhibitors to treat non-small cell lung cancer patients harboring epidermal growth factor receptor mutations

**DOI:** 10.1186/s12890-023-02466-9

**Published:** 2023-05-05

**Authors:** Jinfei Si, Yue Hao, Jingwen Wei, Jing Xiang, Chunwei Xu, Qiuping Shen, Zhengbo Song

**Affiliations:** 1 Department of Clinical Trial, Cancer Hospital of the University of Chinese Academy of Sciences (Zhejiang Cancer Hospital), Hangzhou, 310022 China; 2 The Second Clinical Medical College of Zhejiang Chinese Medical University, Hangzhou, 310053 China; 3 Institute of Basic Medicine and Cancer (IBMC), Chinese Academy of Sciences, Hangzhou, 310022 China; 4 Department of Respiratory Medicine, Jinling Hospital, Nanjing University School of Medicine, Nanjing, China; 5 Tongxiang First People’s Hospital, Tongxiang, 314500 China

**Keywords:** Non-small cell lung cancer (NSCLC), Immune checkpoint inhibitor (ICI), Epidermal growth factor receptor (EGFR), TP53 co-mutation

## Abstract

**Background:**

We aimed to determine the clinical. outcomes of various immune checkpoint inhibitor (ICI) combinations for the treatment of non-small cell lung cancer (NSCLC) patients with epidermal growth factor receptor (EGFR) mutations. The results predicted the treatment efficacy of these combinations.

**Methods:**

From July 15, 2016 to March 22, 2022, 85 NSCLC patients with EGFR mutations, enrolled at the Zhejiang Cancer Hospital, received ICI combinations after resistance to prior EGFR-tyrosine kinase inhibitors (EGFR-TKIs). These patients were diagnosed with EGFR mutations using an amplification refractory mutation system PCR (ARMS-PCR) and next-generation sequencing (NGS). Survival times were analyzed using the Kaplan–Meier method and log-rank test.

**Results:**

Patients who received ICIs combined with anti-angiogenic therapy had longer progression-free survival (PFS) and overall survival (OS) than patients who received ICIs combined with chemotherapy. There was no significant difference in survival time between patients who received ICIs combined with chemotherapy and anti-angiogenic therapy and patients who received ICIs combined with anti-angiogenic therapy or ICIs combined with chemotherapy, which was due to the limitation sample size of patients who received ICIs combined with chemotherapy and anti-angiogenic therapy. Patients with L858R mutations had a longer PFS and OS than patients with exon 19 deletions. T790M negative patients benefited more from ICI combinations, compared with T790M positive patients. In addition, there was no significant difference in PFS and OS between patients with TP53 co-mutations and patients without a TP53 co-mutation. We also found that patients with prior first-generation EGFR-TKI resistance had longer PFS and OS than prior third-generation EGFR-TKI resistance patients. There was no new adverse event in this study.

**Conclusions:**

EGFR-mutated patients who received ICIs combined with anti-angiogenic therapy had longer PFS and OS than patients with ICIs combined with chemotherapy. Patients with L858R or without T790M mutation benefited more from ICI combinations. Besides, patients with prior first-generation EGFR-TKI resistance could benefit more from ICIs combinations than prior third-generation EGFR-TKI resistance patients.

## Introduction

Lung cancer is the leading cause of death worldwide, and non-small cell lung cancer (NSCLC) accounts for more than 80% of lung cancers[1]. Epidermal growth factor receptor (EGFR) mutation has been confirmed as the most common mutation in NSCLC patients. With the rapid development of targeted therapies in recent years, epidermal growth factor receptor-tyrosine kinase inhibitors (EGFR-TKIs) have better clinical benefits than traditional standard chemotherapy in patients with EGFR mutations. NSCLC patients have a median progression-free survival (PFS) of 9–13 months when treated with first- and second-generation EGFR-TKIs, and a median PFS of 18.9 months when treated with third-generation EGFR-TKIs[2–4]. Nevertheless, acquired resistance to EGFR-TKIs is inevitable and the resistance mechanisms are still poorly understood. In addition, therapeutic regimens are limited for patients after EGFR-TKI resistance, due to the poor efficacy of chemotherapy [5].

With the use of immune checkpoint inhibitors (ICIs), which target programmed death receptor-1 (PD-1) and programmed death receptor ligand-1 (PD-L1), the treatment efficacy of ICIs for lung cancer has been confirmed in the CheckMate078 study, OAK study, and Keynote 028 study[6–8]. Consequently, ICIs were approved as first- and second-line treatments for NSCLC patients without driver oncogene alterations. It has been reported that ICI monotherapy did not prolong the survival of NSCLC patients with EGFR mutations, when compared with standard chemotherapy [9]. To overcome these barriers, ICI combinations were recently tested. The IMpower 150 study showed that patients treated with atezolizumab combined with bevacizumab and chemotherapy had better clinical outcomes (PFS, 10.2 months vs. 6.9 months, HR 0.61) and overall survival (OS, not reached vs. 18.7 months, HR 0.61) when compared with patients who received bevacizumab and chemotherapy [10]. In addition, the PROLUNG trial reported that patients with EGFR variations benefited more from pembrolizumab plus docetaxel, when compared with patients who received docetaxel alone, with an overall response rate (ORR) of 58.3% and a median PFS of 6.8 months [11]. Shen et al. analyzed 30 EGFR-mutated patients treated with ICIs alone or in combinations and found that patients who received ICIs combined with chemotherapy had a longer PFS (4.2 vs. 2.9 months) and OS (not reached vs 19.67 months) than ICIs alone[12]. Morimoto et al. enrolled 80 cancer patients with EGFR-mutated lung cancertreated with ICIs alone or chemoimmunotherapy, and found that patients who received chemoimmunotherapy had a longer PFS (5.7 vs. 1.5 months, *P* = 0.001) and OS (18.2 vs. 4.9 months, *P* = 0.001) than patients who received ICIs alone[13]. A multi-center phase II study also reported the promising anti-tumor efficacy of toripalimab plus chemotherapy in NSCLC patients with EGFR mutations [14].

Based on these results, we found that NSCLC patients harboring EGFR mutations who were previously treated with EGFR-TKI could benefit from ICI combinations. Furthermore, the sample sizes of patients with patients with EGFR mutations enrolled in clinical trials and retrospective studies were insufficient, so the treatment efficacy of different ICI combinations remains unclear. This retrospective study therefore aimed to determine the treatment efficacies of various ICI combinations, and to identify potential characteristics that could predict the treatment efficacy of these combinations. To the best of our knowledge, this was the first study with the largest sample size of patients with EGFR mutations, who received ICI combinations.

## Materials and methods

### Patients

We conducted this retrospective study from July 15, 2016 to March 22, 2022, at the Zhejiang Cancer Hospital, involving a cohort of 85 stage IV NSCLC patients harboring EGFR mutations treated with ICIs with progression, after prior EGFR-TKI treatments. We divided 85 NSCLC patients with advanced EGFR mutations into three groups: ICIs combined with a chemotherapy group (*n* = 43), ICIs combined with an anti-angiogenic therapy group (*n* = 23), and ICIs combined with a chemotherapy and anti-angiogenic therapy group (*n* = 19). The ICIs included Nivolumab, Pembrolizumab, Atezolizumab, Tislelizumab, Sintilimab, Camrelizumab, or Toripalimab, and the chemotherapy regimen included platinum-based double chemotherapy, paclitaxel or nab-paclitaxel, and the anti-angiogenic targeted therapy included bevacizumab or anlotinib. Chemotherapy regimens were selected according to NCCN (National Comprehensive Cancer Network) guidelines. The different ICIs treatment strategies were determined by the physician’s clinical experience, the patient’s physical condition, and the medical insurance policy. Besides, a cutoff of 10 months for prior TKI-PFS was set according to previous studies[15]. We also divided all patients into three groups according to prior EGFR-TKI resistance: the 1st EGFR-TKI resistance group, 2nd EGFR-TKI resistance group, and 3rd EGFR-TKI resistance group. Written informed consent was waived due to the nature of the retrospective study. This study was approved by the Ethics Committee of the Zhejiang Cancer Hospital (IRB-2022–160), and adhered to the tenets of the Declaration of Helsinki.

### Detection of gene mutations

Patients in this study were confirmed with NSCLC based on a histopathological examination. EGFR abnormalities, which included deletion of exon 19, a L858R point mutation, a T790M mutation, and other uncommon EGFR mutations were detected by an amplification refractory mutation system PCR (ARMS-PCR) method with the AmoyDx Human EGFR Gene 29 Mutations Detection kit with fluorescence PCR (Amoy Diagnostics, Xiamen, China) and next-generation sequencing (NGS) before receiving ICIs. The exon 19 deletion and L858R mutation were detected by the first ARMS-PCR or NGS, the TP53 mutation was detected by the first NGS, and T790M was detected by the second NGS detection after EGFR-TKI resistance. The gene detection method of NGS followed the College of American Pathologists guidelines [16].

### Statistical analysis

We collected survival data on NSCLC patients enrolled in this study. The endpoints included in this study were ORR, PFS, and OS. The responses of patients with EGFR mutations to ICIs were assessed using the Response Evaluation Criteria in Solid Tumors (RECIST v1.1). ORR was defined as the sum of complete response and partial response. PFS was defined as the time from the date of ICI treatment to disease progression. OS was defined as survival from the date of ICI treatment to death or last follow-up. The survival data of PFS and OS were evaluated using Kaplan–Meier estimates and the log-rank test using Prism 8.0 software for Windows (GraphPad, San Diego, CA, USA). The Cox proportional hazards model was used to identify independent factors that affected the survival times of PFS and OS. Two-sided P-values < 0.05 were defined as statistically significant. Adverse effects were analyzed and graded according to the National Cancer Institute Common Terminology Criteria for Adverse Events, version 5.0. The last follow-up date was July 3, 2022 and the median follow-up time was 14.2 months (range: 1.0–72.0 months).

## Results

### Patient characteristics

A total of 85 advanced NSCLC patients, enrolled at Zhejiang Cancer Hospital from July 15, 2016 to March 22, 2022, and harboring *EGFR* mutations received ICIs for progression after prior EGFR-TKI treatments. The patients were predominantly male, aged < 60 years, not smokers, with a performance status (PS) score of 0–1. There were 83 patients with adenocarcinomas, 10 patients with liver metastases, 13 patients with brain metastases, and 47 patients with bone metastases. Regarding different EGFR subtypes, there were 45 patients with exon 19 deletions, 33 patients with L858R mutations, and seven patients were uncommon EGFR mutations. After resistance to prior EGFR-TKI treatments, there were 26 patients with T790M mutations, 24 patients without T790M mutations, and 35 patients lacking genetic alteration information after EGFR-TKI resistance. In addition, there were 20 patients with TP53 mutations, 21 patients without TP53 mutations, and 44 patients lacking TP53 status information before being treated with ICIs. We also calculated the PFS of patients with prior EGFR-TKI treatments before receiving ICIs. There were 49 patients with TKI-PFS < 10 months and 36 patients ≥ 10 months. There were 44 patients, four patients, and 37 patients resistant to first-, second- and third-generation EGFR-TKI, respectively. All patients enrolled in this study were stage IV with poorly differentiated NSCLCs. The chemotherapy regimens of ICIs combined with chemotherapy only included platinum-based double chemotherapy, and in ICIs combined with chemotherapy and anti-angiogenic therapy, the patients were treated with platinum-based double chemotherapy and paclitaxel or nab-paclitaxel mono-chemotherapy. In addition, the anti-angiogenic-targeted therapy was only treated with bevacizumab. Detailed information of patient characteristics and treatment combinations are listed in Table 1.

**Table 1:** Characteristics of 85 NSCLC patients with EGFR mutations who received ICIs at baseline

Items	Number (%)
Total	85 (100%)
Age	
≤ 60	48 (56.5%)
> 60	37 (43.5%)
Sex	
Male	45 (52.9%)
Female	40 (47.1%)
Smoking History	
Yes	35 (41.2%)
No	50 (58.8%)
PS	
0–1	61 (71.8%)
2	24 (28.2%)
Stage	
IV	85 (100%)
Histologic type	
Squamous carcinoma	2 (2.4%)
Adenocarcinoma	83 (97.6%)
Lines	
2	30 (35.3%)
≥ 3	55 (64.7%)
Liver metastasis	
Yes	10 (11.8%)
No	75 (88.2%)
Brain metastasis	
Yes	13 (15.3%)
No	72 (84.7%)
Bone metastasis	
Yes	47 (55.3%)
No	38 (44.7%)
ICIs treatment Protocols	
ICIs + Chemo	43 (50.6%)
Nivolumab combinations	1 (1.2%)
Pembrolizumab combinations	11 (12.9%)
Camrelizumab combinations	6 (7.1%)
Tislelizumab combinations	4 (4.7%)
Toripalimab combinations	14 (16.5%)
Sintilimab combinations	7 (8.2%)
ICIs + Anti-VEGFR	23 (27.1%)
Pembrolizumab plus bevacizumab	6 (7.1%)
Camrelizumab plus anlotinib	3 (3.5%)
Atezolizumab plus bevacizumab	3 (3.5%)
Toripalimab plus bevacizumab	1 (1.2%)
Toripalimab plus anlotinib	1 (1.2%)
Sintilimab plus bevacizumab	1 (1.2%)
Sintilimab plus anlotinib	8 (9.4%)
ICIs + Chemo + Anti-VEGFR	19 (22.3%)
Pembrolizumab combinations	3 (3.5%)
Camrelizumab combinations	2 (2.4%)
Atezolizumab combinations	7 (8.1%)
Tislelizumab combinations	3 (3.5%)
Toripalimab combinations	2 (2.4%)
Sintilimab combinations	2 (2.4%)
EGFR mutation status	
Exon 19 deletion	45 (53.0%)
L858R mutation	33 (38.8%)
G719X mutation	2 (2.4%)
L861Q mutation	3 (3.4%)
Exon 20 insertion	2 (2.4%)
T790M mutation status	
Positive	26 (30.6%)
Negative	24 (28.2%)
Unknow	35 (41.2%)
TP53 status	
Positive	20 (23.5%)
TP53-19 del co-mutation	8 (9.4%)
TP53-L858R co-mutation	11 (12.9%)
TP53- G719X co-mutation	1 (1.2%)
Negative	21 (24.7%)
Unknow	44 (51.8%)
TKI-PFS	
< 10	49 (57.6%)
≥ 10	36 (42.4%)
Prior EGFR-TKI	
1^st^ EGFR-TKI	
Gefitinib	15 (17.6%)
Erlotinib	2 (2.4%)
Icotinib	27 (31.8%)
2^nd^ EGFR-TKI	
Afatinib	4 (4.7%)
3^rd^ EGFR-TKI	
Osimertinib	37 (43.5%)

*Abbreviations*: *EGFR* Epidermal growth factor receptor, *NSCLC* Non-small cell lung cancer, *ICIs* Immune checkpoint inhibitors, *Chemo* Chemotherapy, *VEGFR* Vascular endothelial growth factor receptor, *TKI-PFS* Tyrosine kinase inhibitors- progression-free survival

### Efficacies of different ICI treatment protocols

**Fig. 1:**
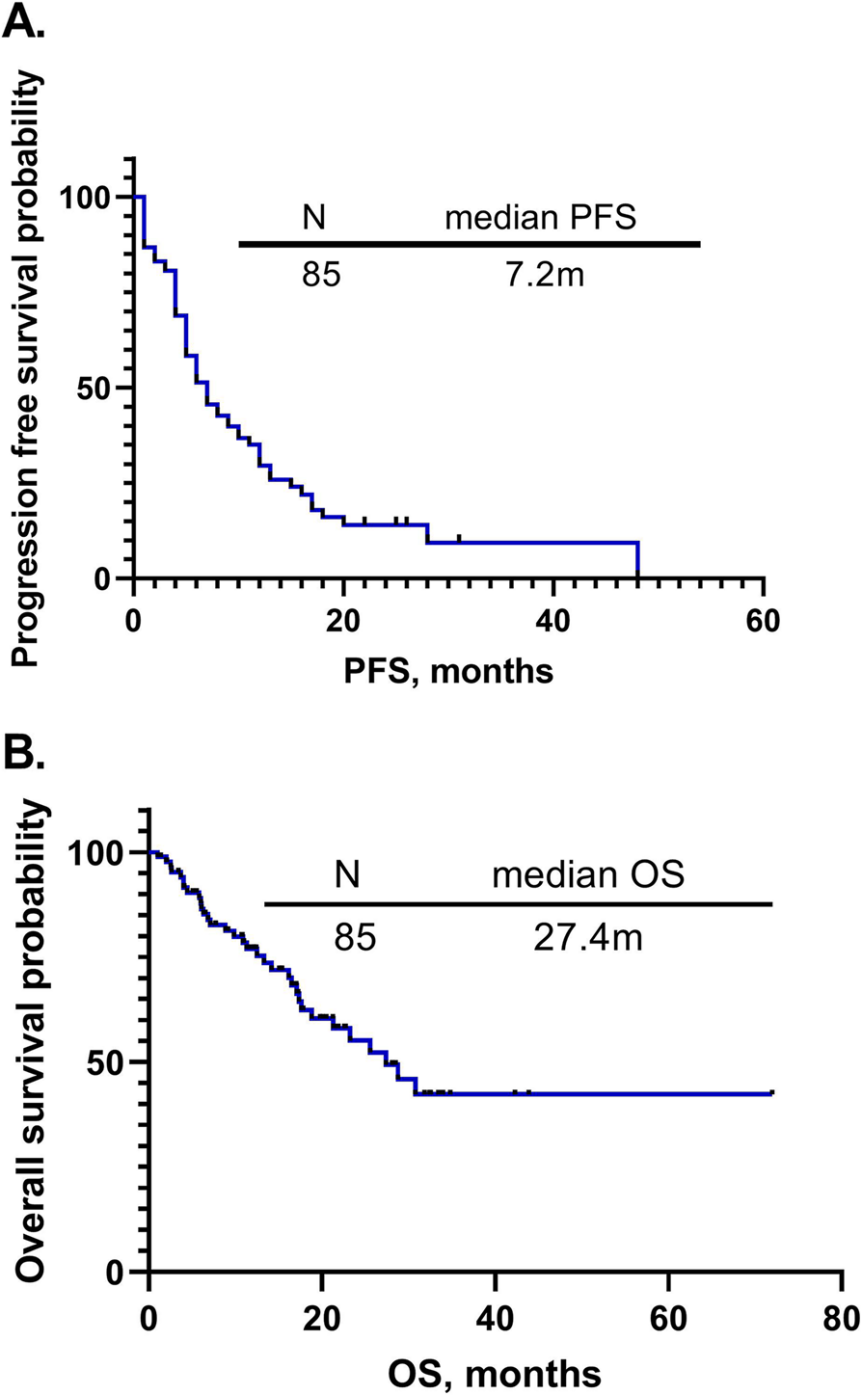
**A** The progression-free survival of 85 non-small cell lung cancer (NSCLC) patients with epidermal growth factor receptor (EGFR) mutations treated with immune checkpoint inhibitor (ICI) combinations. **B** The overall survival of 85 NSCLC patients with EGFR mutations treated with ICI combinations

**Fig. 2:**
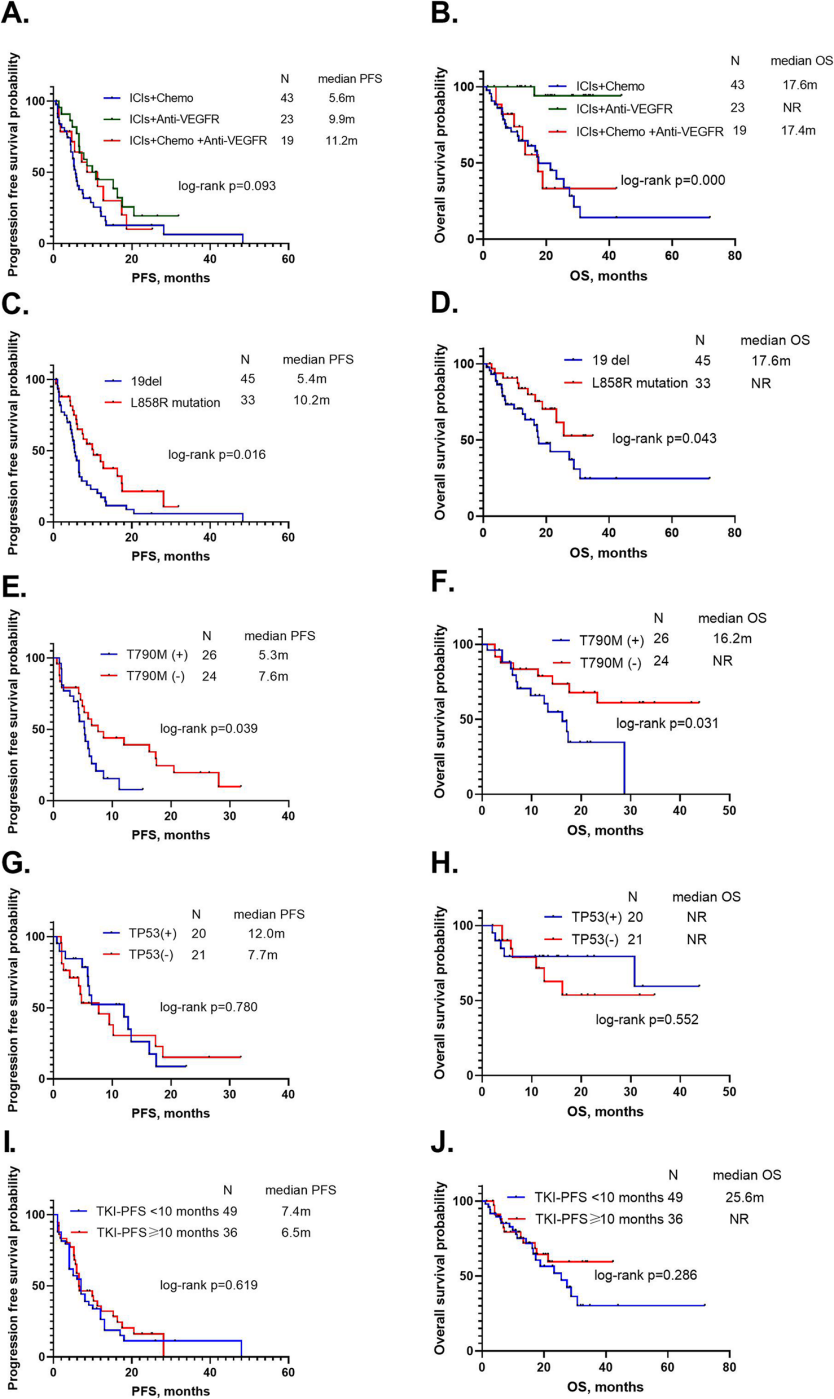
**A** The survival difference in progression-free survival (PFS) between different immune checkpoint inhibitor (ICI) combinations. **B** The survival difference in overall survival (OS) between different ICI combinations. **C** The survival difference in PFS between patients with exon 19 deletions and L858R mutations. **D** The survival difference in OS between patients with exon 19 deletions and L858R mutations. **E** The survival difference in PFS between T790M positive patients and T790M negative patients. **F** The survival difference in OS between T790M positive patients and T790M negative patients. **G** The survival difference in PFS between TP53 co-mutation patients and patients without a TP53 co-mutation. **H** The survival difference in OS between TP53 co-mutation patients and patients without a TP53 co-mutation. **I** The survival difference in PFS between TKI-PFS < 10 months and TKI-PFS > 10 months. **J** The survival difference in OS between TKI-PFS < 10 months and TKI-PFS > 10 months

**Fig. 3:**
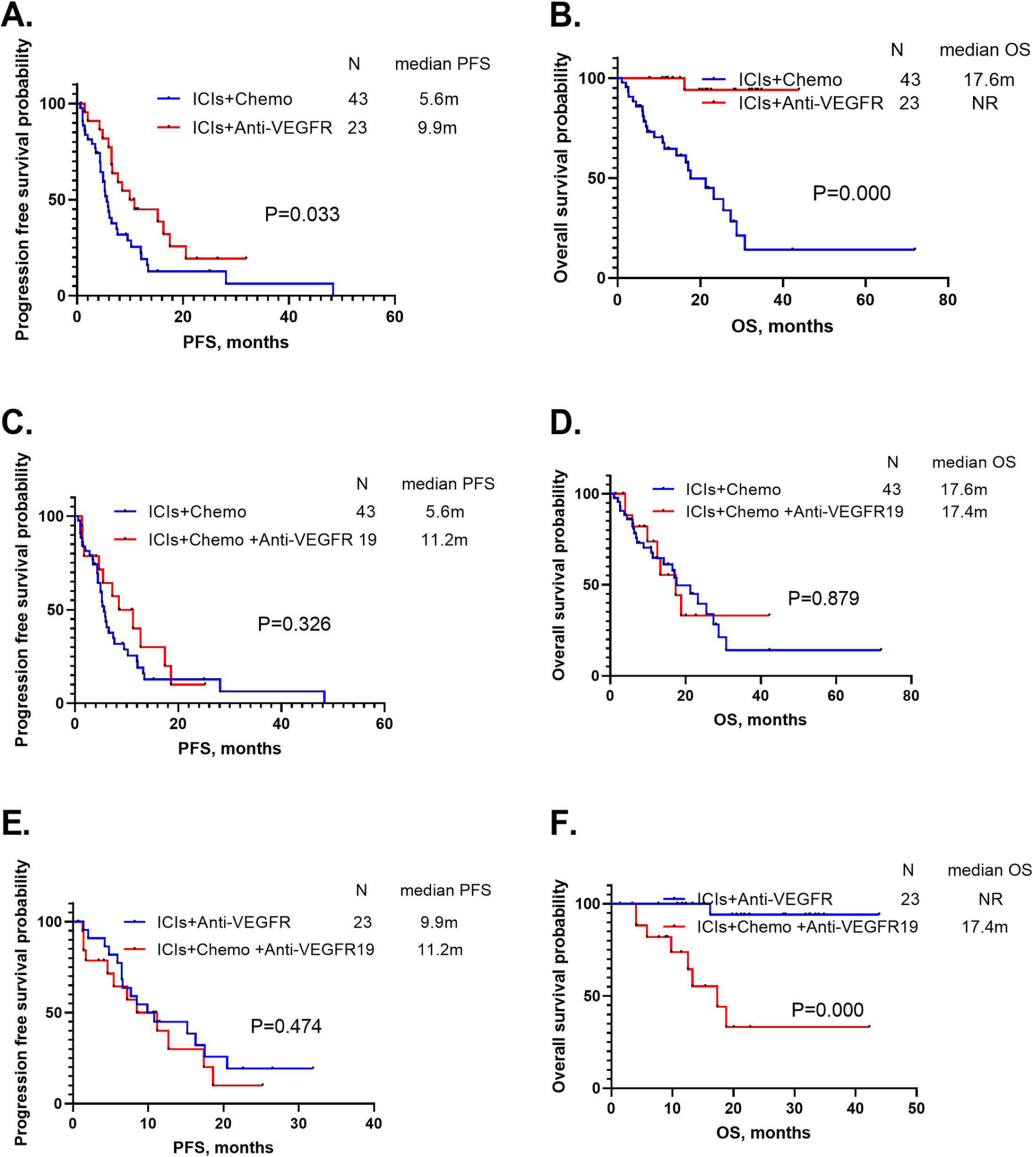
**A** The survival difference in progression-free survival (PFS) between immune checkpoint inhibitors (ICIs) combined with chemotherapy and ICIs combined with anti-angiogenic therapy. **B** The survival difference in overall survival (OS) between ICIs combined with chemotherapy and ICIs combined with anti-angiogenic therapy. **C** The survival difference in PFS between ICIs combined with chemotherapy and ICIs combined with chemotherapy and anti-angiogenic therapy. **D** The survival difference in OS between ICIs combined with chemotherapy and ICIs combined with chemotherapy and anti-angiogenic therapy. **E** The survival difference in PFS between ICIs combined with anti-angiogenic therapy and ICIs combined with chemotherapy and anti-angiogenic therapy. **F** The survival difference in OS between ICIs combined with anti-angiogenic therapy and ICIs combined with chemotherapy and anti-angiogenic therapy

Among 85 advanced NSCLC patients with EGFR mutations who received ICIs, the ORR, median PFS, and OS were 21.2%, 7.2 months [95% confidence interval (CI): 5.6–8.9, Fig. 1A] and 27.4 months (95% CI:19.2–35.6, Fig. 1B), respectively. The best response of patients with EGFR mutations to ICI combinations was PR in this study. We next divided them into three groups: the ICIs combined with the chemotherapy group (*n* = 43), the ICIs combined with the anti-angiogenic therapy group (*n* = 23), and the ICIs combined with the chemotherapy and anti-angiogenic therapy group (*n* = 19), then compared the survival differences between them. The ORR, median PFS, and OS of the ICIs combined with chemotherapy group were 27.9%, 5.6 months (95% CI: 4.8–6.5) and 17.6 months (95% CI: 11.2–24.1), respectively. The ORR, median PFS, and OS of the ICIs combined with anti-angiogenic therapy group were 8.7%, 9.9 months (95% CI: 5.4–14.4) and not reached, respectively. The ORR, median PFS, and OS of the ICIs combined with chemotherapy and anti-angiogenic therapy group were 21.1%, 11.2 months (95% CI: 5.0–17.3), and 17.4 months (95% CI:6.5–28.2), respectively. There was no significant difference in PFS between the groups (*P* = 0.093), but there was a significant difference in OS (*P* = 0.000) (Fig. 2A-B) (Table 2). We next compared the survival differences and found that patients treated with ICIs combined with anti-angiogenic therapy had longer PFS and OS than patients who received ICIs combined with chemotherapy (*P* = 0.033 and *P* = 0.000, respectively) (Fig. 3A-B). There was no significant difference in PFS (*P* = 0.326) and OS (*P* = 0.879) between patients who received ICIs combined with chemotherapy and anti-angiogenic therapy and ICIs combined with chemotherapy (Fig. 3C-D). Patients treated with ICIs combined with chemotherapy and anti-angiogenic therapy had a shorter OS (*P* = 0.000) and similar PFS (*P* = 0.474) than patients with ICIs combined with anti-angiogenic therapy (Fig. 3E-F).

**Table 2:** Survival analyses of 85 NSCLC patients with EGFR mutations treated with ICIs

Items	N	ORR	PFS (95%CI)	*P*	OS (95%CI)	*P*
Total	85	21.2%	7.2 (5.6–8.9)	–	27.4 (19.2–35.6)	–
ICIs + Chemo	43	27.9%	5.6 (4.8–6.5)		17.6 (11.2–24.1)	
ICIs + Anti-VEGFR	23	8.7%	9.9 (5.4–14.4)	0.093	NR	0.000
ICIs + Chemo + Anti-VEGFR	19	21.1%	11.2 (5.0–17.3)		17.4 (6.5–28.2)	
Exon 19 deletion	45	20%	5.4 (4.3–6.4)	0.016	17.6 (12.0–23.3)	0.043
L858R mutation	33	24.2%	10.2 (5.2–15.2)		NR	
T790M mutation	26	19.2%	5.3 (3.9–6.7)	0.039	16.2 (10.8–21.6)	0.031
Non-T790M mutation	24	16.7%	7.6 (3.6–11.6)		NR	
TP53 positive	20	35.0%	12.0 (2.1–21.9)	0.780	NR	0.552
TP53 negative	21	19.0%	7.7 (2.2–13.3)		NR	
Prior TKI-PFS < 10 months	49	28.6%	7.4 (5.2–9.7)	0.619	25.6 (14.3–36.9)	0.286
Prior TKI-PFS ≥ 10 months	36	11.1%	6.5 (2.2–10.8)		NR	
1^st^ EGFR-TKI resistance	44	25%	12.0 (8.3–15.7)	0.000	NR	0.001
3^rd^EGFR-TKI resistance	37	13.6%	5.2 (4.4–6.0)		16.2 (19.1–35.7)	

*Abbreviations*: *EGFR* Epidermal growth factor receptor, *NSCLC* Non-small cell lung cancer, *ICIs* Immune checkpoint inhibitors, *Chemo* Chemotherapy, *VEGFR* Vascular endothelial growth factor receptor, *TKI-PFS* Tyrosine kinase inhibitors- progression-free survival

### Efficacy of ICIs for EGFR subtypes and TP53 mutations

Among 85 NSCLC patients with EGFR mutations treated with ICIs, there were 45 patients with exon 19 deletions, 33 patients with L858R mutations, and seven patients with uncommon EGFR mutations. The ORR, median PFS, and OS of patients with exon 19 deletions were 20%, 5.4 months (95% CI: 4.3–6.4), and 17.6 months (95% CI: 12.0–23.3), respectively. The ORR, median PFS, and OS of patients with L858R mutations were 24.2%, 10.2 months (95% CI: 5.2–15.2), and not reached, respectively. There was a remarkable difference between them in PFS (*P* = 0.016) and OS (*P* = 0.043), and patients with L858R mutations had a longer PFS and OS than patients with exon 19 deletions (Fig. 2C-D) (Table 2).

After progression following prior EGFR-TKI treatments, 50 patients had information regarding T790M mutation, with 26 with T790M mutations and 24 without T790M mutations. The ORR, median PFS, and OS of patients with T790M mutations were 19.2%, 5.3 months (95% CI: 3.9–6.7), and 16.2 months (95% CI: 10.8–21.6), respectively. The ORR, median PFS, and OS of patients without T790M mutations were 16.7%, 7.6 months (95% CI: 3.6–11.6), and not reached, respectively. There was a significant difference between the groups in PFS (*P* = 0.039) and OS (*P* = 0.031), and patients without T790M mutation had a longer PFS and OS (Fig. 2E-F) (Table 2).

We also collected data on patients with or without TP53 co-mutations before they received ICIs, and found 20 patients with TP53 co-mutations and 21 patients without a TP53 co-mutation. The ORR, median PFS, and OS of patients with TP53 co-mutations were 35.0%, 12.0 months (95% CI: 2.1–21.9), and not reached, respectively. The ORR, median PFS, and OS of patients without TP53 co-mutations were 19.0%, 7.7 months (95% CI: 2.2–13.3), and not reached, respectively. There was no significant difference in PFS (*P* = 0.780) and OS (*P* = 0.552) between them (Fig. 2G-H) (Table 2).

### Effects of the previous EGFR-TKI and TKI-PFS on ICI efficacies

**Fig. 4:**
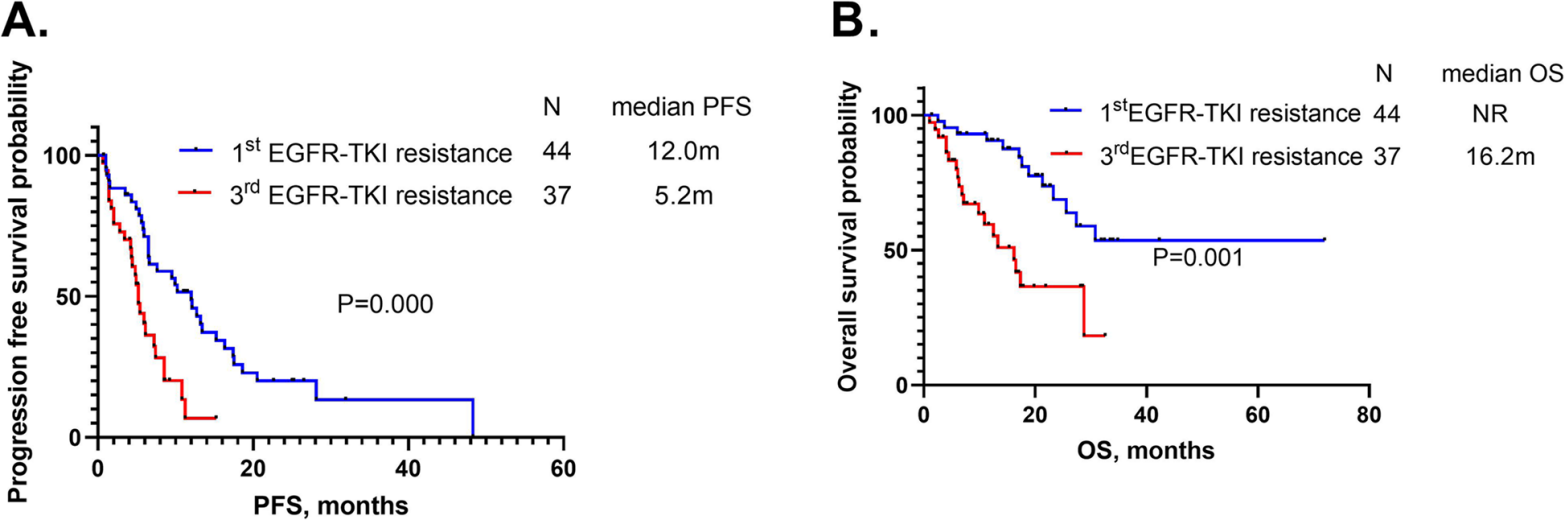
**A** Comparison of progression-free survival (PFS) between the groups categorized according to prior EGFR-TKI resistance. **B** Comparison of overall survival (OS) between the groups categorized according to prior EGFR-TKI resistance

All patients enrolled in this study had disease progression after prior EGFR-TKI treatments. We divided all patients into two groups: 1^st^ EGFR-TKI resistance group and 3^rd^ EGFR-TKI resistance group. The 2^nd^ EGFR-TKI resistance group was not analyzed due to the small quantity. The ORR, median PFS and OS of 1^st^ EGFR-TKI resistance group were 25%, 12.0 months (95%CI:8.5–15.7) and not reached. The ORR, median PFS and OS of 3^rd^ EGFR-TKI resistance group were 13.6%, 5.2 months (95%CI:4.4–6.0) and 16.2 months (95%CI:19.1–35.7), respectively. We found that 1^st^ EGFR-TKI resistance group had longer PFS (*P* = 0.000) and OS than 3^rd^ EGFR-TKI resistance group (*P* = 0.001) (Fig. 4A-B).

Next, we calculated the PFS of the most recent EGFR-TKI treatment before patients received ICIs, and divided them into two groups: the PFS < 10 months group (*n* = 49) and the PFS ≥ 10 months group (*n* = 36). The ORR, median PFS, and OS of the PFS below 10 months group were 28.6%, 7.4 months (95% CI: 5.2–9.7), and 25.6 months (95% CI: 14.3–36.9), respectively. The ORR, median PFS, and OS of the PFS over 10 months group were 11.1%, 6.5 months (95% CI: 2.2–10.8), and not reached, respectively There was no significant difference between them in PFS (*P* = 0.619) and OS (*P* = 0.286) (Fig. 2I-J) (Table 2).

### Independent prognostic factors affecting PFS and OS

**Fig. 5:**
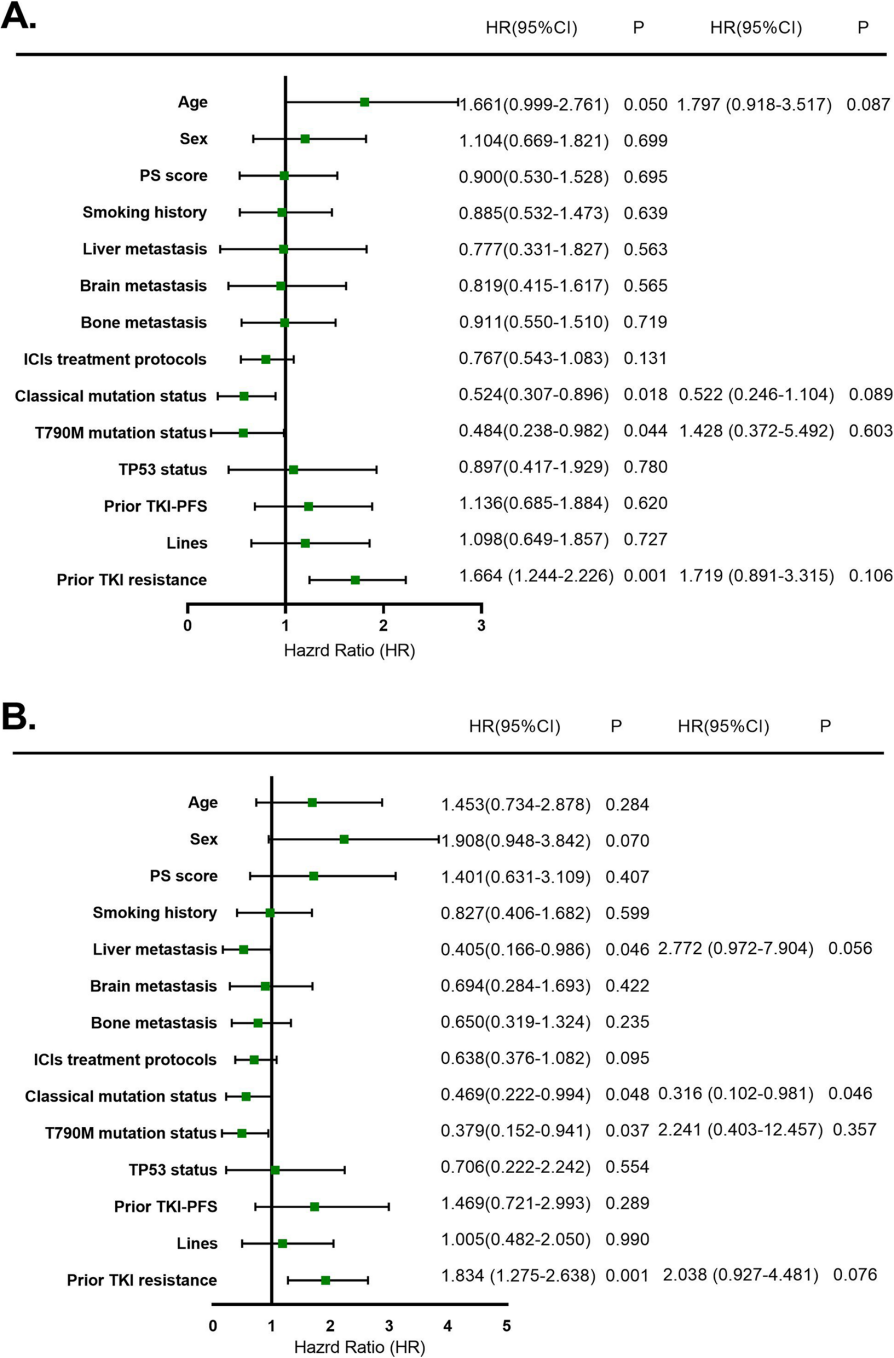
**A** Cox regression analysis of factors associated with progression-free survival in 85 non-small cell lung carcinoma (NSCLC) patients with epidermal growth factor receptor (EGFR) mutations treated with immune checkpoint inhibitor (ICI) combinations. **B** Cox regression analysis of factors associated with overall survival in 85 NSCLC patients with EGFR mutations treated with ICI combinations

We established the Cox proportional hazard regression model in 85 NSCLC patients with EGFR mutations who were treated with ICIs, to determine independent prognostic factors affecting the PFS and OS. We found that age [hazard ratio (HR): 1.661; 95% CI: 0.999–2.761, *P* = 0.050), classical mutation status, which included exon 19 deletions and L858R mutations (HR: 0.524; 95% CI: 0.307–0.896, *P* = 0.018), T790M mutation status (HR: 0.484; 95% CI: 0.238–0.982, *P* = 0.044) and prior EGFR-TKI resistance (HR:1.664; 95%CI:1.244–2.226, *P* = 0.001) partially affected the PFS using univariable analysis. We used multivariable analysis to show that these four factors did not affect the PFS (*P* = 0.175, *P* = 0.116, and *P* = 0.229), respectively (Fig. 5A).

We also used the Cox proportional hazard regression model of OS to show that liver metastasis (HR: 0.405; 95% CI: 0.166–0.986, *P* = 0.046), classical mutation status, which included exon 19 deletions and L858R mutations (HR: 0.469; 95% CI: 0.222–0.994, *P* = 0.048), T790M mutation status (HR: 0.379; 95% CI: 0.152–0.941, *P* = 0.037) and prior EGFR-TKI resistance (HR:1.834; 95%CI:1.275–2.638, *P* = 0.001) affected OS using univariable analysis. In addition, classical mutation status (HR:0.316; 95%CI:0.102–0.981, *P* = 0.046) still affected OS using multivariable analysis, while the other two factors did not affect the OS (*P* = 0.103 and *P* = 0.370) (Fig. 5B). Liver metastasis was also an independent prognostic factor affecting OS.

### Adverse events of patients treated with ICI combinations

In this study, the most common adverse events of ICIs combinations were anemia (51.8%), leukopenia (36.5%), hypertension (31.8%), nausea and vomiting (30.6%), and thyroid dysfunction (30.6%). The most common adverse events of grade 3 were neutropenia (4.7%), leukopenia (3.5%), and anemia (2.3%). In the ICIs combined with chemotherapy group, the most common adverse events were anemia (62.8%), leukopenia (44.2%), and neutropenia (37.2%). Grade 3 adverse events included neutropenia (7.0%), leukopenia (4.7%), and anemia (2.3%). The most common adverse events in the ICIs combined with anti-angiogenic therapy group were hypertension (60.9%) and thyroid dysfunction (43.5%), and in the grade 3 event it was thrombocytopenia (4.3%). In addition, the most common adverse events in the ICIs combined with anti-angiogenic and chemotherapy therapy group were anemia (57.9%), nausea and vomiting (47.4%), and thyroid dysfunction (47.4%), while the grade 3 adverse events included neutropenia (5.3%), leukopenia (5.3%), anemia (5.3%), ALT increases (5.3%), and AST increases (5.3%) (Table 3). These results showed that nausea and vomiting were more common in the ICIs combined with anti-angiogenic and chemotherapy therapy group, hypertension was more common in the ICIs combined with anti-angiogenic therapy group, and hematological toxicities, including leukopenia, neutropenia, and anemia were more common in the ICIs combined with chemotherapy group.

**Table 3:** Adverse events of 85 NSCLC patients with EGFR mutations treated with ICIs

Adverse Events	Total	ICIs + Chemo		ICIs + Anti-VEGFR	ICIs + Chemo + Anti-VEGFR		
	N (%)	Any grade	Grade 3	Any grade	Grade 3	Any grade	Grade 3
Fatigue	8 (9.4%)	5 (11.6%)	0 (0%)	2 (8.7%)	0 (0%)	1 (5.3%)	0 (0%)
Nausea/vomiting	26 (30.6%)	12 (27.9%)	0 (0%)	5 (21.7%)	0 (0%)	9 (47.4%)	0 (0%)
Constipation	5 (5.9%)	1 (2.3%)	0 (0%)	2 (8.7%)	0 (0%)	2 (10.5%)	0 (0%)
Hypertension	27 (31.8%)	5 (11.6%)	0 (0%)	14 (60.9%)	0 (0%)	8 (42.1%)	0 (0%)
Rash	7 (8.2%)	2 (4.7%)	0 (0%)	4 (17.4%)	0 (0%)	1 (5.3%)	0 (0%)
Leukopenia	31 (36.5%)	19 (44.2%)	2 (4.7%)	6 (26.1%)	0 (0%)	6 (31.6%)	1 (5.3%)
Neutropenia	24 (28.2%)	16 (37.2%)	3 (7.0%)	3 (13.0%)	0 (0%)	5 (26.3%)	1 (5.3%)
Thrombocytopenia	12 (14.1%)	6 (14.0%)	0 (0%)	4 (17.4%)	1 (4.3%)	2 (10.5%)	0 (0%)
Anaemia	44 (51.8%)	27 (62.8%)	1 (2.3%)	6 (26.1%)	0 (0%)	11 (57.9%)	1 (5.3%)
ALT increase	12 (14.1%)	6 (14.0%)	0 (0%)	3 (13.0%)	0 (0%)	3 (15.8%)	1 (5.3%)
AST increase	12 (14.1%)	7 (16.3%)	0 (0%)	3 (13.0%)	0 (0%)	2 (10.5%)	1 (5.3%)
Creatinine increase	11 (12.9%)	4 (9.3%)	0 (0%)	5 (21.7%)	0 (0%)	2 (10.5%)	0 (0%)
Thyroid dysfunction	26 (30.6%)	7 (16.3%)	0 (0%)	10 (43.5%)	0 (0%)	9 (47.4%)	0 (0%)
Proteinuria	6 (7.1%)	4 (9.3%)	0 (0%)	0 (0%)	0 (0%)	2 (10.5%)	0 (0%)
Pneumonia	1 (1.2%)	1 (2.3%)	0 (0%)	0 (0%)	0 (0%)	0 (0%)	0 (0%)

*Abbreviations*: *EGFR* Epidermal growth factor receptor, *NSCLC* Non-small cell lung cancer, *ICIs* Immune checkpoint inhibitors, *Chemo* Chemotherapy, *VEGFR* Vascular endothelial growth factor receptor

## Discussion

In this study, EGFR-mutated patients benefited more from ICIs combined with anti-angiogenic therapy than from ICIs combined with chemotherapy. Patients with L858R mutations or T790M negative patients benefited more from ICI combinations. There was no difference between patients with or without TP53 co-mutations. In addition, patients with prior first-generation EGFR-TKI resistance could benefit more from ICIs combinations than third-generation EGFR-TKI resistance patients. To the best of our knowledge, this had the largest sample size of patients with EGFR mutations treated with ICI combinations. In this study, we determined the treatment efficacies of different ICIs combinations. In addition, we analyzed subgroups of patients with EGFR mutations that could benefit more from ICIs combinations, and we also determined the role of TP53 co-mutations, prior EGFR-TKI resistance and prior TKI-PFS in predicting treatment efficacies of ICIs combinations. This study provides a theoretical basis for individualized precision medicine for cancer.

Patients who received ICIs combined with anti-angiogenic therapy had a longer PFS and OS than patients with ICIs combined with chemotherapy. According to CheckMate 012, the median PFS and OS of six patients with EGFR mutations treated with nivolumab plus chemotherapy were 4.8 months and 20.5 months, respectively [17]. A multi-center phase II study reported that the ORR and median PFS of 40 patients with EGFR mutations who received toripalimab combined with chemotherapy were 50% and 7.0 months, respectively[14]. In the IMpower150 study, the sample size of EGFR-positive patients receiving ICI combinations was 79, and the ORR, median PFS, and OS of patients receiving atezolizumab plus bevacizumab plus chemotherapy were 70.6%, 10.2 months, and not estimated, respectively. In addition, the ORR, median PFS, and OS of patients treated with atezolizumab plus chemotherapy were 35.6%, 6.9 months, and 21.4 months, respectively[10]. We obtained similar outcomes and found that the treatment regimen of ICIs combined with anti-angiogenic therapy was a better choice than ICIs combined with chemotherapy. However, ICIs combined with anti-angiogenic therapy had a lower ORR while showing superior PFS when compared to ICIs combined with chemotherapy in our study. This phenomenon could be explained by the small sample size of EGFR mutated patients received the ICIs combinations regimens, especially ICIs combined with anti-angiogenic therapy, which may influence the results to some extent.

The efficacy difference between ICIs combined with chemotherapy and anti-angiogenic therapy and ICIs combined with anti-angiogenic therapy or ICIs combined with chemotherapy remained unclear and needs to be validated in the future. Furthermore, the results of this study showed patients harboring L858R mutations benefited more from ICI combinations, when compared with patients with exon 19 deletions. Hastings et al. enrolled 171 patients, who received ICIs, and found that patients with L858R mutations had a higher response and longer OS than patients harboring exon 19 deletions, with an ORR and median OS of 15.2% and 12.1 months, respectively. They also reported that patients with exon 19 deletions had a lower tumor mutation burden (TMB), when compared with patients with L858R mutations, which was associated with responses to ICIs [18]. Jin et al. studied 20 patients with EGFR mutations, who received ICIs, and reported that patients with L858R mutations had higher PD-L1 expression and were positively associated with an inflammatory phenotype [19]. Bai et al. conducted a retrospective study, which included 75 patients with ECFR mutations who were treated with ICIs, and reported that patients harboring L858R mutations tended to benefit more from ICIs alone or in combination, when compared with patients with exon 19 deletions, although the difference was not significant (PFS: 3.9 vs. 3.87 months; OS: 13.2 vs. 7.07 months, respectively) [20]. We obtained similar results, which showed that patients with L858R mutations had a significantly longer PFS and OS than patients with exon 19 deletions.

In the present study, patients without T790M mutations also had a longer PFS and OS than patients with T790M mutations who received ICIs combinations. A previous study by Haratani et al. enrolled 25 patients with EGFR mutations, who received nivolumab, and reported that T790M negative patients tended to benefit more from ICIs than T790M positive patients, after failure from prior EGFR-TKI treatments, although the difference in PFS was not significant (2.1 vs. 1.3 months, P = 0.099) [21]. Yamada et al. enrolled 27 patients with EGFR mutations and found that T790M negative patients had a longer PFS than T790M positive patients (86 vs. 48 days, P = 0.03) [22]. Shen et al. analyzed 30 patients with EGFR mutations, who were treated with ICIs alone or in combinations, and found that T790M negative patients had a longer PFS (4.23 vs. 1.70 months) and OS (28.53 vs. 10.17 months) than T790M positive patients (P = 0.019 and P = 0.014), respectively [12]. We obtained similar outcomes. Nevertheless, the Cox regression models showed that EGFR subtypes and T790M mutation status were not independent prognostic factors for the PFS and OS. To the best of our knowledge, patients with exon 19 deletions tended to acquire T790M mutations, when compared with L858R mutations, after resistance to prior EGFR-TKI treatments [23]. In the present study, the incidences of T790M mutations in patients with exon 19 deletions and L858R mutations after EGFR-TKI resistance were 46.7% (21/45) and 15.2% (5/33), respectively. To a limited extent, there was a causal relationship between them, which was the reason why the Cox regression model did not show a prognostic role of EGFR subtypes and T790M mutations.

In this study, there was no survival difference between patients with TP53 co-mutations and patients without TP53 co-mutations, when they received ICI combinations. Wang et al. studied 42 patients with EGFR mutations who received ICIs, and found that TP53 positive patients had a longer PFS than patients without a TP53 mutation (6.7 vs. 2.6 months, P = 0.003)[24]. The PD-L1 and TMB biomarkers could therefore be used to predict treatment efficacies of ICIs. However, there is an urgent need for novel biomarkers that predict the efficacies of ICIs. For this reason, the predictive role of TP53 co-mutations in immunotherapy remains unclear and needs further validation.

Patients with prior first-generation EGFR-TKI resistance could benefit more from ICIs combinations than third-generation EGFR-TKI resistance patients. Besides, there was no significant difference in PFS and OS between patients with prior TKI-PFS shorter than 10 months and longer than 10 months. Fang et al. carried out a translational study and showed that EGFR-TKI treatment would remodel the tumor immune microenvironment in EGFR-mutated patients[25]. Isomoto et al. enrolled 138 EGFR-mutated patients; they demonstrated that the proportion of patients with high PD-L1 expression level (≥ 50%) were increased and the tumor mutation burden was higher than before after EGFR-TKI treatment[26]. The above data showed that EGFR-TKI treatment could remodel the tumor microenvironment and therefore could benefit from ICIs. However, the clinical data which prior EGFR-TKI resistance could benefit more from ICIs was lacking. In this study, we found that EGFR-mutated patients with prior first-generation EGFR-TKI resistance could benefit more from ICIs combinations than third-generation EGFR-TKI resistance patients.

Liu et al. studied 58 patients with EGFR mutations, who were treated with ICIs, and reported that patients with TKI-PFS < 10 months had a longer PFS (15.1 vs. 3.8 months, *P* = 0.0002) and higher ORR (31.8% vs. 10%) than patients with TKI-PFS > 10 months, who were treated with ICIs [27]. In contrast, Bai et al. studied 75 patients with EGFR mutations, who received ICIs, and reported that patients with a TKI-PFS > 10 months tended to have a longer PFS than patients with a TKI-PFS < 10 months (5.2 vs, 2.8 months, *P* = 0.005) [20]. However, we found different outcomes. We enrolled 85 patients harboring EGFR mutations with ICI combinations, and we found that there was no significant difference in PFS and OS between patients with a TKI-PFS < 10 months and > 10 months. Controversy over the predictive role of prior TKI-PFS patients treated with ICIs, therefore needs further study.

This study had some limitations. First, the sample size of ICI combinations used to treat patients harboring EGFR mutations was insufficient and needed to be expanded in the future. Second, the retrospective nature of our study could have influenced the results. Third, the ICI combinations and chemotherapy regimens were heterogeneous. Fourth, the OS data in this study were too early because ICI combinations were only recently used in clinical practice. Further research is therefore necessary to confirm our results.

## Conclusions

In this study, ICIs combined with anti-angiogenic therapy was a better choice than ICIs combined with chemotherapy for NSCLC patients with EGFR mutations. Patients with L858R mutations or T790M negative patients benefited more from ICI combinations. Patients with prior first-generation EGFR-TKI resistance could benefit more from ICIs combinations than prior third-generation EGFR-TKI resistance patients. In addition, TP53 co-mutation as a predictive factor for patients with EGFR mutations, who received ICI combinations, remains unclear. Furthermore, there was no adverse event in this study.

## Data Availability

The full data and materials can be obtained from Zhengbo Song upon sufficient and reasonable request.

## Abbreviations

ICI: Immune checkpoint inhibitor
NSCLC: Non-small cell lung cancer
EGFR: Epidermal growth factor receptor
EGFR-TKIs: Epidermal growth factor receptor-tyrosine kinase inhibitors
ARMS-PCR: Amplification refractory mutation system PCR
NGS: Next-generation sequencing
PFS: Progression-free survival
OS: Overall survival
PD-1: Programmed death receptor-1
PD-L1: Programmed death receptor ligand-1
ORR: Overall response rate
NCCN: National Comprehensive Cancer Network
RECIST: Response Evaluation Criteria in Solid Tumors
PS: Performance status
